# A multiscale computational model of arterial growth and remodeling including Notch signaling

**DOI:** 10.1007/s10237-023-01697-3

**Published:** 2023-04-06

**Authors:** Jordy G.M. van Asten, Marcos Latorre, Cansu Karakaya, Frank P.T. Baaijens, Cecilia M. Sahlgren, Tommaso Ristori, Jay D. Humphrey, Sandra Loerakker

**Affiliations:** 1 Department of Biomedical Engineering, Eindhoven University of Technology, Eindhoven, The Netherlands; 2 Institute for Complex Molecular Systems, Eindhoven University of Technology, Eindhoven, The Netherlands; 3 Center for Research and Innovation in Bioengineering, Universitat Politècnica de València, València, Spain; 4 Faculty of Science and Engineering, Biosciences, Åbo Akademi, Turku, Finland; 5 Department of Biomedical Engineering, Yale University, New Haven, CT, USA

**Keywords:** Constrained mixture model, Notch signaling, Growth and remodeling, Mechanobiology, Artery, Jagged ligands

## Abstract

Blood vessels grow and remodel in response to mechanical stimuli. Many computational models capture this process phenomenologically, by assuming stress homeostasis, but this approach cannot unravel the underlying cellular mechanisms. Mechano-sensitive Notch signaling is well-known to be key in vascular development and homeostasis. Here, we present a multiscale framework coupling a constrained mixture model, capturing the mechanics and turnover of arterial constituents, to a cell−cell signaling model, describing Notch signaling dynamics among vascular smooth muscle cells (SMCs) as influenced by mechanical stimuli. Tissue turnover was regulated by both Notch activity, informed by in vitro data, and a phenomenological contribution, accounting for mechanisms other than Notch. This novel framework predicted changes in wall thickness and arterial composition in response to hypertension similar to previous in vivo data. The simulations suggested that Notch contributes to arterial growth in hypertension mainly by promoting SMC proliferation, while other mechanisms are needed to fully capture remodeling. The results also indicated that interventions to Notch, such as external Jagged ligands, can alter both the geometry and composition of hypertensive vessels, especially in the short term. Overall, our model enables a deeper analysis of the role of Notch and Notch interventions in arterial growth and remodeling and could be adopted to investigate therapeutic strategies and optimize vascular regeneration protocols.

## 1 Introduction

Blood vessels grow and remodel in response to changing mechanical stimuli. It is generally hypothesized that this phenomenon serves to establish and maintain mechanical homeostasis (Humphrey 2008). Such mechano-regulated growth and remodeling (G&R) has been captured by numerous computational models using one of two basic approaches (reviewed in Cyron and Humphrey (2017); Ambrosi et al. (2019)). Kinematic growth models emphasize consequences of growth by describing changes in the shape and size of a body (Rodriguez et al. 1994); constrained mixture models (CMMs) focus more on processes of G&R by accounting for the production and removal of individual tissue constituents having distinct mechanical properties and stress-free configurations in evolving tissue states (Humphrey and Rajagopal 2002). CMMs have been successful in capturing the associated mechanobiology and similarly immunobiology, thus increasing our understanding of vascular G&R in health, disease, and regeneration. For example, they have been adopted to predict how the alignment of new collagen fibers affects the stability of enlarging intracranial aneurysms (Baek et al. 2006). Conversely, they have predicted important features of the in vivo development of tissue-engineered vessels from polymeric scaffolds and enabled the systematic assessment of scaffold parameters (Miller et al. 2015; Drews et al. 2020). In most cases, these CMMs have described tissue production and removal using phenomenological constitutive relations, not accounting directly for the cellular mechanisms underlying mechano-regulated G&R (e.g., Baek et al. 2006, 2007; Valentin et al. 2009, 2011; Latorre et al. 2019; Latorre and Humphrey 2020). Notwithstanding the insights gained using this approach, phenomenological modeling does not allow direct investigation of the consequences of pathological mutations or targeted interventions. Including more detailed mechanistic behavior of cells will enhance the modeling capabilities. For this reason, cell signaling models have been coupled with tissue-level G&R models to yield more mechanistic understanding of the G&R (Virag et al. 2015, 2023; Aparicio et al. 2016; Marino et al. 2017; Keshavarzian et al. 2018; Irons et al. 2021, 2022). Such models demand significant information on the many parallel signaling pathways, which is often not available. An alternate strategy, therefore, is to augment phenomenological G&R models with details on single signaling pathways that are particularly important in particular processes.

One of the main pathways responsible for vascular development and homeostasis is juxtacrine Notch signaling among the vascular smooth muscle cells (SMCs) within the medial layer (Iso et al. 2003; Gridley 2007; Baeten and Lilly 2017). In the canonical Notch pathway, Jagged or Delta-like ligands interact with membrane-bound Notch receptors of adjacent cells, resulting in the cleavage of the Notch intracellular domain (NICD), which then translocates to the nucleus to regulate the transcription of Notch target genes. Numerous studies have revealed that Notch strongly influences the behavior of SMCs in health and disease (Iso et al. 2003; Gridley 2007; Del Monte et al. 2011; Baeten and Lilly 2017; Mašek and Andersson 2017; Li and Kong 2020; Ristori et al. 2021; Karakaya et al. 2022b), including during many instances of G&R. The Notch pathway is also increasingly recognized as mechano-sensitive (Stassen et al. 2020, Karakaya and Van Asten et al. 2022a). For example, Notch signaling between SMCs responds to changes in strain by altering the expression of Notch receptors and Jagged ligands (Morrow et al. 2005; Loerakker et al. 2018; Karakaya et al. 2022a). This mechano-sensitivity, combined with its influence on SMC behavior, makes the Notch pathway a strong candidate to be one of the key mechanisms responsible for mechano-regulated G&R in blood vessels.

Several computational models of Notch signaling have been developed to increase our understanding of Notch-regulated processes in various tissues (Binshtok and Sprinzak 2018). The mechano-sensitivity of Notch has also been included in recent models to predict the phenotype of SMCs (Loerakker et al. 2018; Ristori et al. 2020; Van Asten and Ristori et al. 2022). Simulations with these models have suggested that Notch mechano-sensitivity may be one of the key mechanisms in both the establishment (Loerakker et al. 2018) and maintenance (Van Asten and Ristori et al. 2022) of arterial homeostasis, given its role in modulating the SMC phenotype. For example, we have recently adopted this model to show that the sensitivity of Notch to mechanical strain at mean blood pressure may explain, in part, the thickening of the arterial wall in response to hypertension (Van Asten and Ristori et al. 2022). Yet, the tissue-scale G&R necessary for modeling homeostasis was only implied, not modeled explicitly. Thus, it was not possible to investigate the full feedback cycle involving Notch signaling, tissue-level G&R, and wall mechanics to simulate the establishment and maintenance of arterial homeostasis and to understand the role of Notch in these processes.

Here, we develop a multiscale computational framework combining Notch signaling dynamics with tissue-level G&R to understand better the role of Notch signaling in the mechano-regulated G&R of blood vessels. Coupling the Notch model to a CMM extends the capabilities of these individual models, enabling us to explore effects of Notch interventions and study the role of Notch in long-term vascular homeostasis. This coupling was informed by in vitro data obtained from human coronary artery SMCs. G&R was assumed to depend not only on Notch, but also on a combination of other factors, grouped together in a phenomenological contribution. The multiscale framework was first tested by simulating hypertensive arteries and subsequently adopted to investigate effects of interventions to the Notch pathway. This application was motivated by previous studies suggesting that Notch could serve as a therapeutic target in vascular disease (Zhou et al. 2015; Zhu et al. 2017; Davis et al. 2018; Morris et al. 2019; Ristori et al. 2021) and regeneration (Carlson et al. 2007; Zohorsky and Mequanint 2021; Tiemeijer and Ristori et al. 2022a; Karakaya and Van Asten et al. 2022a, Tiemeijer and Sanlidag 2022b). The model predicted that Notch mainly contributes to arterial thickening in response to hypertension by promoting SMC proliferation and that other mechanisms are necessary to fully capture remodeling. The simulations further suggested that presenting external Jagged ligands to SMCs primarily affects the arterial composition in the early stages of adaptation. Taken together, our model is a step forward in including mechano-sensitive Notch signaling in a computational G&R framework and may serve as a tool to predict the effects of Notch and Notch interventions on arterial G&R.

## 2 Methods

### 2.1 Constrained mixture model

Following the theory of constrained mixtures (Humphrey and Rajagopal 2002; Latorre and Humphrey 2018a), the arterial wall was modeled as a continuum consisting of the structurally significant constituents collagen, elastin, and SMCs. The model accounts for the distinct rates of turnover of these constituents, as well as their individual material properties and natural (i.e., stress-free) configurations. Once the constituents are deposited, they are assumed to deform with the mixture. In this section, we discuss the evolving kinematics, mass densities, the strain energy functions, the stresses, and the constituent-specific behaviors of this model. The CMM formulation presented here is based on Latorre et al. 2018b, which was adapted to model the arterial wall as a single layer. The novelty lies in the coupling of this CMM to a computational Notch model (Sect. 2.2) to account for Notch-regulated tissue production (see Sect. 2.4).

#### 2.1.1 Kinematics

**Fig. 1:**
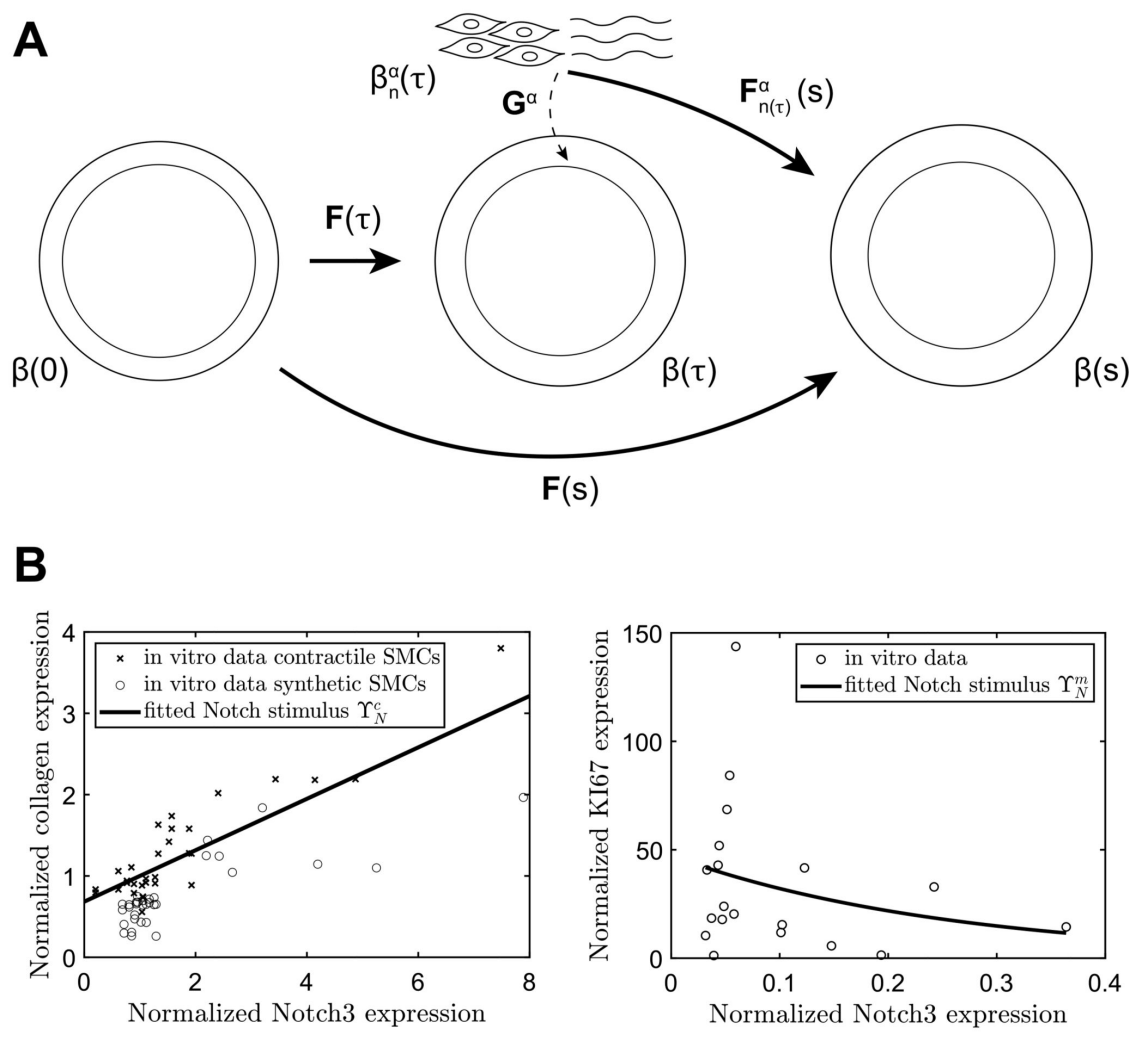
(**A**) Schematic representation of the evolving arterial geometry in the CMM. (**B**)qPCR data correlating Notch3 gene expression to collagen gene expression (left) and KI67 gene expression (right) in human coronary artery SMCs. The data on collagen expression were obtained from both synthetic and contractile SMCs, while the data on KI67 expression were obtained from synthetic SMCs. All values were normalized to the geometric mean of the contractile group to obtain relative expression values. The lines indicate the fits through the data, used as the Notch stimulus functions for collagen $\left({ϒ}_{N}^{c}\right)$ and SMCs $\left({ϒ}_{N}^{m}\right)$ in Eqs. (32) and (33)

Figure 1A shows the evolving configurations of the mixture at three G&R times. Configuration *β*(0), at G&R time *s* = 0, is taken as the homeostatic reference configuration, while *β*(*s*) is the configuration at the current G&R time *s*. The configurations at intermediate times *τ* are denoted as *β*(*τ*), with 0 ≤ *τ* ≤ *s*. Figure 1A also shows the natural configuration of constituents *α* deposited at time *τ* labeled as $\beta_{n}^{\alpha }\left(\tau \right)$, with *α* = *c* for collagen, *α* = *m* for SMCs, and *α* = *e* for elastin. Deformations of the mixture are captured by the deformation gradient tensor **F**. The constituents are deposited into the mixture with constituent-specific deposition stretches **G**^*α*^, which were assumed to be constant and symmetric. The deformation of a constituent *α*, which was deposited at time *τ* from its natural configuration to the configuration of the mixture at time *s* is then expressed as:

(1) $${\text{F}}_{n\left(\tau \right)}^{\alpha }\left(s\right)=\text{F}\left(s\right)={\text{F}}^{-1}\left(\tau \right){\text{G}}^{\alpha }.$$

The associated right Cauchy-Green tensor is defined as:

(2) $${\text{C}}_{n\left(\tau \right)}^{\alpha }\left(s\right)={\text{F}}_{n\left(\tau \right)}^{\alpha T}\left(s\right){\text{F}}_{n\left(\tau \right)}^{\alpha }\left(s\right),$$

where superscript *T* denotes the transpose. Equation (2) may be rewritten as:

(3) $${\text{C}}_{n\left(\tau \right)}^{\alpha }\left(s\right)={\text{G}}^{\alpha }{\text{F}}^{-T}\left(\tau \right)\text{C}\left(s\right){\text{F}}^{-1}\left(\tau \right){\text{G}}^{\alpha }$$

with **C**(*s*)=**F**^*T*^(*s*)**F**(*s*).

#### 2.1.2 Mass density and strain energy evolution

Constituents may be produced or removed during the G&R process. The evolution of the referential mass density of each constituent (i.e., constituent mass per unit reference volume of the mixture), is given by:

(4) $$\rho_{R}^{\alpha }\left(s\right)=\int_{-\infty }^{s}m_{R}^{\alpha }\left(\tau \right)q^{\alpha }\left(s,\tau \right)d\tau ,$$

where $m_{R}^{\alpha }\left(\tau \right)$ is the referential rate of mass density production of constituent *α* at time *τ*, while *q^α^*(*s, τ*) is a survival function indicating the fraction of constituent *α* deposited at time *τ* that is still present at time *s*. The initial referential mass densities are calculated as $\rho_{R}^{\alpha }\left(0\right)={\text{Φ}}_{o}^{\alpha }\rho$, where ${\text{Φ}}_{o}^{\alpha }$ are the initial constituent mass fractions and *ρ* is the mass density of the mixture. We followed the common assumption (Latorre and Humphrey 2018a) that this density is constant and coincides with the true mass densities of each of the constituents (i.e., their current mass per current volume). Similarly, the evolution of the strain energy function per unit reference volume of the mixture can be written for each constituent as:

(5) $$W_{R}^{\alpha }\left(s\right)=\frac{1}{\rho }\int_{-\infty }^{s}m_{R}^{\alpha }\left(\tau \right)q^{\alpha }\left(s,\tau \right){\hat{W}}^{\alpha }\left(C_{n\left(\tau \right)}^{\alpha }\left(s\right)\right)d\tau ,$$

where ${\hat{W}}^{\alpha }$ is the strain energy function per unit volume of constituent *α*.

#### 2.1.3 Stresses

The time scale of tissue G&R is assumed to be much longer than that of the tissue’s mechanical response. Consequently, while the volume of the tissue can change between G&R time points, the tissue is assumed to be incompressible during transient deformations due to loading at each fixed G&R time. The Cauchy stress of the mixture is therefore given by:

(6) $$\sigma \left(s\right)=\sum_{\alpha }\sigma^{\alpha }\left(s\right)-p\left(s\right)I,$$

with *σ^α^* the Cauchy stress of constituent *α* and *p*(*s*) a Lagrange multiplier associated with the incompressibility constraint, which needs to be determined from the equilibrium equations and boundary conditions.

From the strain energy function in Eq. (5), we can derive the second Piola−Kirchhoff stress for each constituent at the mixture level, namely:

(7) $${\text{S}}^{\alpha }\left(s\right)=2\frac{\partial W_{R}^{\alpha }\left(s\right)}{\partial \text{C}\left(s\right)}.$$

We rewrite Eq. (7) using the chain rule as:

(8) $${\text{S}}^{\alpha }\left(s\right)=\frac{2}{\rho }\int_{-\infty }^{s}m_{R}^{\alpha }\left(\tau \right)q^{\alpha }\left(s,\tau \right)\frac{\partial {\hat{W}}^{\alpha }\left({\text{C}}_{n\left(\tau \right)}^{\alpha }\left(s\right)\right)}{\partial {\text{C}}_{n\left(\tau \right)}^{\alpha }\left(s\right)}:\frac{\partial {\text{C}}_{n\left(\tau \right)}^{\alpha }\left(s\right)}{\partial \text{C}\left(s\right)}d\tau .$$

To determine the derivative in the last term of Eq. (8), we rewrite the right Cauchy-Green tensor from Eq. (3) as:

(9) $${\text{C}}_{n\left(\tau \right)}^{\alpha }\left(s\right)={\text{G}}^{\alpha }{\text{F}}^{-T}\left(\tau \right)⊙{\text{G}}^{\alpha }{\text{F}}^{-T}\left(\tau \right):\text{C}\left(s\right),$$

where the symbol ⊙ denotes the crossed dyadic product, (**A** ⊙ **B**)_*ijkl*_ = *A_ik_B_jl_*, and find:

(10) $$\frac{\partial {\text{C}}_{n\left(\tau \right)}^{\alpha }\left(s\right)}{\partial \text{C}\left(s\right)}={\text{G}}^{\alpha }{\text{F}}^{-T}\left(\tau \right)⊙{\text{G}}^{\alpha }{\text{F}}^{-T}\left(\tau \right).$$

Substituting this expression into Eq. (8) and rearranging, we obtain:

(11) $${\text{s}}^{\alpha }\left(s\right)=\frac{1}{\rho }\int_{-\infty }^{s}m_{R}^{\alpha }\left(\tau \right)q^{\alpha }\left(s,\tau \right){\text{F}}^{-1}\left(\tau \right){\text{G}}^{\alpha }{\hat{\text{S}}}^{\alpha }\left({\text{C}}_{n\left(\tau \right)}^{\alpha }\left(s\right)\right){\text{G}}^{\alpha }{\text{F}}^{-T}\left(\tau \right)d\tau ,$$

where we have introduced the second Piola−Kirchhoff stress at the constituent level (derived from ${\hat{W}}^{\alpha }$), defined as:

(12) $${\hat{\text{S}}}^{\alpha }\left({\text{C}}_{n\left(\tau \right)}^{\alpha }\left(s\right)\right)=2\frac{\partial {\hat{W}}^{\alpha }\left({\text{C}}_{n\left(\tau \right)}^{\alpha }\left(s\right)\right)}{\partial {\text{C}}_{n\left(\tau \right)}^{\alpha }\left(s\right)}.$$

By performing the usual push-forward operation, we obtain the Cauchy stress tensor at the mixture level:

(13) $$\sigma^{\alpha }\left(s\right)=\frac{1}{J\left(s\right)}\text{F}\left(s\right){\text{S}}^{\alpha }\left(s\right){\text{F}}^{T}\left(s\right),$$

with the volume ratio *J*(*s*)= det**F**(*s*). Using Eq. (11), we obtain:

(14) $$\sigma^{\alpha }\left(s\right)=\frac{1}{\rho J\left(s\right)}\int_{-\infty }^{s}m_{R}^{\alpha }\left(\tau \right)q^{\alpha }\left(s,\tau \right)\text{F}\left(s\right){\text{F}}^{-1}{\text{G}}^{\alpha }{\hat{\text{S}}}^{\alpha }\left({\text{C}}_{n\left(\tau \right)}^{\alpha }\left(s\right)\right){\text{G}}^{\alpha }{\text{F}}^{-T}{\text{F}}^{T}\left(s\right)d\tau .$$

Finally, using Eq. (1), this reduces to:

(15) $$\sigma^{\alpha }\left(s\right)=\frac{1}{\rho J\left(s\right)}\int_{-\infty }^{s}m_{R}^{\alpha }\left(\tau \right)q^{\alpha }\left(s,\tau \right){\text{F}}_{n\left(\tau \right)}^{\alpha }\left(s\right){\hat{\text{S}}}^{\alpha }\left({\text{C}}_{n\left(\tau \right)}^{\alpha }\left(s\right)\right){\text{F}}_{n\left(\tau \right)}^{\alpha T}\left(s\right)d\tau .$$

#### 2.1.4 Constituent-specific behavior

Next, constitutive relations for the hyperelastic response, rate of mass density production, and survival of each of the constituents need to be prescribed. As elastin (*α* = *e*) to their natural configurations. Substituting this expression is mainly produced during development and degraded very into Eq. (12) and applying the chain rule, we obtain the slowly, we adopted the common assumption that elastin does contributions of collagen and SMCs to the second not turn over during a hypertensive G&R process. As such, Piola−Kirchhoff stress at the constituent level: the referential mass density of elastin (Eq. (4)) simplifies to:

(16) $$\rho_{R}^{e}\left(s\right)=\rho_{R}^{e}\left(0\right)=:\rho_{R}^{e}$$

and the referential strain energy density of elastin at the mixture level (Eq. (5)) becomes:

(17) $$W_{R}^{e}\left(s\right)=\frac{\rho_{R}^{e}}{\rho }{\hat{W}}^{e}\left({\text{C}}^{e}\left(s\right)\right).$$

A neo-Hookean function was assumed for the elastin-dominated amorphous material at the constituent level:

(18) $${\hat{W}}^{e}\left({\text{C}}^{e}\left(s\right)\right)=\frac{c^{e}}{2}\left({\text{C}}^{e}\left(s\right):\text{I}-3\right),$$

with *c^e^* the shear modulus. We obtain the following second Piola−Kirchhoff stress at the constituent level (analogous to Eq. (12) with ${\text{C}}^{e}\left(s\right)\equiv {\text{C}}_{n\left(0\right)}^{e}\left(s\right)$):

(19) $${\hat{\text{S}}}^{e}\left({\text{C}}^{e}\left(s\right)\right)=2\frac{\partial {\hat{W}}^{e}\left({\text{C}}^{e}\left(s\right)\right)}{\partial {\text{C}}^{e}\left(s\right)}c^{e}\text{I}$$

Substituting Eq. (19) into Eq. (15) and simplifying using Eqs. (16) and (17) gives the Cauchy stress contribution of elastin:

(20) $$\sigma^{e}=\frac{\rho^{e}R^{C^{e}}}{\rho }\text{F}\left(s\right){\text{G}}^{e}{\text{G}}^{e}{\text{F}}^{T}\left(s\right),$$

with $\text{F}\left(s\right){\text{G}}^{e}{=\text{F}}^{e}\left(s\right)\equiv {\text{F}}_{n\left(0\right)}^{e}\left(s\right)$ representing the deformation of elastin from its natural configuration to the current configuration (analogous to ${\text{F}}_{n\left(\tau \right)}^{\alpha }\left(s\right)$ in Eq. (1)).

We considered 2 families of collagen fibers in a symmetric helical arrangement, similar to previous studies (Holzapfel et al. 2000; 2002; Baek et al. 2006), at an angle of *ϕ* with respect to the axial direction. In addition, similar to prior studies (Humphrey et al. 2007; Valentin and Humphrey, 2009; Latorre and Humphrey 2018a, b), we assumed that the SMCs are oriented in the circumferential direction and only contribute to the stress in that direction. Following previous studies (Latorre and Humphrey 2018a, b), a Fung-type constitutive function was selected to describe the hyperelastic response of collagen and SMCs:

(21) $${\hat{W}}^{\alpha }\left(\lambda_{n\left(\tau \right)}^{\alpha }\left(s\right)\right)=\frac{c_{1}^{\alpha }}{4c_{2}^{\alpha }}\left(\exp\left[c_{2}^{\alpha }{\left(\lambda_{n\left(\tau \right)}^{\alpha 2}\left(s\right)-1\right)}^{2}\right]-1\right)$$

where *α* = *c, m* for collagen and SMCs, respectively, $c_{1}^{\alpha }$ and $c_{2}^{\alpha }$ are constituent-specific material parameters and $\lambda_{n\left(\tau \right)}^{\alpha }\left(s\right)$ represents the stretch at time s of the collagen fibers or SMCs deposited at time *τ* in their respective directions with respect to their natural configurations. Substituting this expression into Eq. (12) and applying the chain rule, we obtain the contributions of collagen and SMCs to the second Piola−Kirchhoff stress at the constituent level:

(22) $$\begin{array}{c} {\hat{\text{S}}}^{\alpha }\left({\text{C}}_{n\left(\tau \right)}^{\alpha }\left(s\right)\right)=2\frac{\partial {\hat{W}}^{\alpha }\left(\lambda_{n\left(\tau \right)}^{\alpha 2}\left(s\right)\right)}{\partial \lambda_{n\left(\tau \right)}^{\alpha 2}\left(s\right)}\frac{\partial \lambda_{n\left(\tau \right)}^{\alpha 2}\left(s\right)}{\partial {\text{C}}_{n\left(\tau \right)}^{\alpha }\left(s\right)}=c_{1}^{\alpha }\left(\lambda_{n\left(\tau \right)}^{\alpha 2}-1\right)\\ \exp\left[c_{2}^{\alpha }{\left(\lambda_{n\left(\tau \right)}^{\alpha 2}-1\right)}^{2}\right]{\text{a}}^{\alpha }⊗{\text{a}}^{\alpha } \end{array}$$

where **a**^*α*^ is the unit vector of the direction of constituent *α*, defined in the stress-free configuration $\beta_{n}^{\alpha }\left(\tau \right)$. Equation (22) can be substituted into Eq. (15) to find the corresponding Cauchy stresses. Similar to previous bio-chemo-mechanical models of vascular G&R (Aparicio et al. 2016; Marino et al. 2017; Keshavarzian et al. 2018, 2019; Irons and Humphrey 2021), the active stress generated by SMCs was not considered in the present model.

Removal of constituents is modeled with the following exponential survival function:

(23) $$q^{\alpha }\left(s,\tau \right)=\exp\left(-\int_{\tau }^{s}k^{\alpha }\left(t\right)dt\right),$$

with *k^α^* the degradation rate given by:

(24) $$k^{\alpha }\left(t\right)=k_{o}^{\alpha }\left(1+{\left(\text{Δ}\sigma \left(t\right)\right)}^{2}\right),$$

where Δ*σ* indicates the deviation of pressure- and axial force-induced intramural stress from a homeostatic target:

(25) $$\text{Δ}\sigma \left(t\right)=\frac{\tilde{\sigma }\left(t\right)-{\tilde{\sigma }}_{o}}{{\tilde{\sigma }}_{o}}.$$

Here, $\tilde{\sigma }$ represents a scalar measure of the intramural stress (defined in Sect. 2.4) and subscript o denotes the original value.

The referential rate of production of the constituents, $m_{R}^{\alpha }$, is regulated by a stimulus function ϒ^*α*^:

(26) $$m_{R}^{\alpha }\left(\tau \right)=m_{N}^{\alpha }\left(\tau \right){ϒ}^{\alpha }\left(\tau \right)=k^{\alpha }\left(\tau \right)\rho_{R}^{\alpha }\left(\tau \right){ϒ}^{\alpha }\left(\tau \right)$$

where the (generally evolving) nominal production rate $m_{N}^{\alpha }$ can be written in terms of the degradation rate *k^α^* and the referential mass density $\rho_{R}^{\alpha }$. Classically, the stimulus function ϒ^*α*^ is determined phenomenologically and depends on deviations in stress from homeostatic target values. In the present study, ϒ^*α*^ is partly determined mechanistically: in addition to deviations in stress, ϒ^*α*^ also depends on the Notch signaling activity in the SMCs. Thus, the definition of ϒ^*α*^ will be given in more detail in Sect. 2.4, after describing the Notch signaling model.

### 2.2 Notch signaling model

A previously developed model for cell−cell Notch signaling (Loerakker et al. 2018) was used herein to inform the tissue-scale G&R in the CMM. This model is based on prior studies (Sprinzak et al. 2011; Boareto et al. 2015), and has proven useful in investigating roles of Notch in hypertensive remodeling independent of tissue-level G&R (Van Asten and Ristori et al. 2022). Briefly, the model considers a one-dimensional array of SMCs in the radial direction of the arterial wall. Previous computational analysis from our group indicated that this one-dimensional array is sufficient to represent the signaling occurring in 2D and 3D in arteries (Ristori et al. 2020). The content of several Notch-related proteins is tracked in each SMC individually using the following set of rate equations (ordinary differential equations):

(27) $$\begin{array}{c} \frac{dN_{j}}{dt}=N_{pr}\exp\left(A_{N}E_{\theta \theta }\right)H^{S}\left(I_{j},\Lambda ,p\right)-k_{c}N_{j}\left(D_{j}+J_{j}\right)\\ -k_{t}N_{j}\left(\frac{D_{j-1}+D_{j+1}+J_{j-1}+J_{j+1}}{2}\right)-k_{t}N_{j}\left(J_{sol}+J_{im}\right)-\gamma N_{j} \end{array}$$

(28) $$\frac{dJ_{j}}{dt}=J_{pr}\exp\left(A_{J}E_{\theta \theta }\right)H^{S}\left(I_{j},\Lambda ,p\right)-k_{c}J_{j}N_{j}-k_{t}J_{j}\left(\frac{N_{j-1}+N_{j+1}}{2}\right)-\gamma J_{j}$$

(29) $$\frac{dD_{j}}{dt}=D_{pr}H^{S}\left(I_{j},\Lambda ,p\right)-k_{c}D_{j}N_{j}-k_{t}D_{j}\left(\frac{N_{j-1}+N_{j+1}}{2}\right)-\gamma D_{j}$$

(30) $$\frac{dI_{j}}{dt}=k_{t}N_{j}\left(\frac{D_{j-1}+D_{j+1}+J_{j-1}+J_{j+1}}{2}\right)+k_{t}N_{j}J_{im}-\gamma_{I}I_{j}$$

where *N_j_, J_j_, D_j_*, and *I_j_* represent the content of Notch, Jagged, Delta, and NICD in cell *j*, respectively, and *k_t_* and *k_c_* are rate parameters for trans-activation (between receptors and ligands of adjacent cells) and cis-inhibition (between receptors and ligands of the same cell), respectively. The parameters *J_sol_* and *J_im_* represent the number of soluble and immobilized Jagged ligands available per SMC, respectively. The autoregulatory effects of Notch activation on the production of Notch, Jagged and Delta, are included by multiplying the base production rates of these proteins (*N_pr_, J_pr_*, and *D_pr_*) with a Hill function accounting for the Notch transcriptional activity following trans-activation:

(31) $$H^{S}\left(I,\Lambda ,p\right)=\Lambda +\frac{1-\Lambda }{1+{\left(I/I_{0}\right)}^{p}},$$

where Λ determines the changes in protein production due to trans-activation, *I*_0_ defines the transition point between a convex and a concave response of the Hill function, and *p* indicates how sensitive the protein production is to the NICD content. The mechano-sensitivity of Notch and Jagged in VSMCs (Morrow et al. 2005; Loerakker et al. 2018) is included by modulating the production of these proteins using an exponential function dependent on the normal Green−Lagrange strain of the SMCs in the circumferential direction, *E_θθ_*, and on the mechano-sensitivity parameters *A_N_* and *A_J_* for Notch and Jagged, respectively. Protein degradation is described with the degradation parameters *γ* and *γ_I_*.

Analogous to a previous approach (Tiemeijer and Ristori et al. 2022a), Eqs. (27) and (30) were modified from earlier works (Loerakker et al. 2018; van Engeland et al. 2019; Ristori et al. 2021; Van Asten and Ristori et al. 2022) to incorporate effects of external Jagged ligands, either soluble (*J_sol_*) or immobilized to a surface (*J_im_*). In the current model, Notch receptors not only can interact with cell-bound Jagged and Delta ligands, but also with external Jagged ligands (Eq. (27)). Based on experimental observations, it was assumed that interactions between Notch receptors and immobilized Jagged ligands lead to Notch activation and release of NICD (Beckstead et al. 2009; Bhattacharyya et al. 2014; Manokawinchoke et al. 2017), while soluble Jagged ligands inhibit Notch signaling by occupying Notch receptors without activating them (Caolo et al. 2011; Xiao et al. 2013; Zhou et al. 2015). As a result, binding between Notch and *J_im_* could result in Notch activation and consequential increase in NICD in Eq. (30) (second term), while binding between Notch and *J_sol_* only elicited a decrease in the free Notch content (Eq. 27, fourth term). It was assumed that the external ligands have the same affinity to Notch as native, cell-bound ligands. As the interaction rate *k_t_* depends on the receptor-ligand affinity (Luca et al. 2017), the interactions between Notch receptors and external Jagged ligands were assumed to occur at the same *k_t_* as trans-interactions. *k_t_* 2017). Furthermore, a constant content of external Jagged ligands was assumed by assigning constant values to *J_sol_* and *J_im_*. This assumption was made as we are interested in the effects of external Jagged when they still have a significant contribution to G&R, and not when their concentration has diminished over time.

### 2.3 Experimental data

To correlate tissue formation with different levels of Notch activity in the model, data from in vitro experiments, obtained in our previous study (Karakaya et al. 2022b), were analyzed and used as input. The methods for cell culture, induction of Notch signaling, and qPCR analysis are described in detail in our previous work (Karakaya et al. 2022b). Briefly, human coronary artery SMCs (Lonza) were cultured for a minimum of 7 days, according to the manufacturer’s protocol, in human vascular muscle cell basal medium (Gibco) supplemented with either 5% smooth muscle growth supplement (Gibco) to obtain synthetic SMCs or 1% smooth muscle differentiation supplement (Gibco) to obtain contractile SMCs. Data from contractile and synthetic cells were used to correlate Notch activity with collagen synthesis, while only data from synthetic cells were used to correlate Notch activity with proliferation. Cell-culture plates were coated with 2.2 μg/cm^2^ of bovine fibronectin (Thermo Fisher Scientific). For Notch signal activation, 50 μg/ml Recombinant Protein G (Thermo Fisher Scientific) was added to the fibronectincoated plates, followed by the immobilization of 2 μg/ml Recombinant Human Jagged1-Fc Chimera Protein (R and D systems) to Protein G. Synthetic and contractile SMCs were subsequently seeded on either fibronectin-coated or Jagged1-Fc immobilized plates, and cultured for 3 days in their corresponding media. Samples were collected in RLT buffer, and RNA was isolated using the RNeasy mini kit (Qiagen) according to the manufacturer’s protocol. 165 ng of RNA was used to synthesize cDNA with a reaction including 50 ng random primers (Promega), 10 mM dNTPs (Invitrogen), 5 × first strand buffer (Invitrogen), 0.1 M DTT (Invitrogen) and M-MLV Reverse Transcriptase (Invitrogen). qPCR was run on a CFX 384 Thermal Cycler (Bio-Rad) with iQ SYBR Green Supermix (Bio-Rad). Ct values of *COL1A1, COL3A1, KI67*, and *NOTCH3* were normalized for the housekeeping gene *B2M* (PrimerDesign). The resulting data were normalized to the geometric mean of the contractile group and calculated with comparative C_T_ method to obtain relative expression values (Schmittgen and Livak 2008).

### 2.4 Coupling and implementation

In the present study, a multiscale framework was established by coupling the CMM for arterial G&R to the Notch signaling model. The coupling was bi-directional, with information from the CMM serving as input for the Notch model, and vice versa. The strain-sensitivity of Notch (Eqs. (27) and (28)) enabled straightforward coupling from the CMM to the Notch model. Specifically, the Green−Lagrange strains of the SMCs in circumferential direction were used as input for the Notch model, defined as: $E_{\theta \theta }=\frac{1}{2}\left(\lambda_{\theta }^{2}-1\right)$, where $\lambda_{\theta }:=\lambda_{n\left(\tau \right)}^{m}\left(s\right)$ is the SMC stretch corresponding to the deformation ${\text{F}}_{n\left(\tau \right)}^{m}\left(s\right)$ in Fig. 1A. For simplicity, the stretches from the cohorts of SMCs, deposited at times *τ*, were averaged by dividing the sum of these stretches by the number of cohorts. We included only the 2000 most recently deposited cohorts because older cohorts were assumed not to contribute significantly to the stretch as most of their material was already removed. In addition, the thickness *h* calculated after each time step in the CMM was used to update the number of SMCs, *M*, in the radial array of SMCs in the Notch model. In particular, *M* was computed as *h*/*h_c_* where *h*_c_ is the SMC thickness assumed to be 2 μm. Thus, *M* was determined independent of the SMC density. This approach was chosen for simplicity as determining *M* as a function of the SMC density would require more uncertain assumptions about the 3D SMC arrangement. This simplification is not expected to influence the results as previous analysis has shown that the predicted Notch activity was not significantly affected by the number of SMC layers (Ristori et al. 2020). The value of *h_c_* was based on the initial number of SMCs in radial direction: *M_o_* = 16, estimated from histological data (Lacolley et al. 2017), and the initial thickness: *h_o_* = 0.032 mm (Bersi et al. 2017).

To couple the Notch model to the CMM, the influence of Notch activity on the rate of mass production of collagen and SMC proliferation was accounted for by introducing Notch stimulus functions for SMCs $\left({ϒ}_{N}^{m}\right)$ and collagen $\left({ϒ}_{N}^{c}\right)$. These functions were fitted to the in vitro data correlating Notch3 expression to both collagen expression and SMC proliferation. SMC proliferation was quantified by the expression of the proliferation marker KI67 (Sect. 2.3 and Fig. 1B). Notch3 expression was taken as a measure for Notch activity, given the known upregulation of this gene in response to Notch activation in SMCs (Loerakker et al. 2018). In the model, Notch activity was represented by the NICD content *I*. This resulted in the following functions fitted to the in vitro data, visualized in Fig. 1B:

(32) $${ϒ}_{N}^{m}=47.21e^{-3.855I}$$

(33) $${ϒ}_{N}^{c}=0.3161\left(I-1\right)+1$$

normalized such that ${ϒ}_{N}^{m}\left(I=1\right)={ϒ}_{N}^{c}\left(I=1\right)=1$. These correlations assume that the measured changes in collagen and KI67 expression were a result of only changes in Notch signaling. In the case of KI67, this is motivated by the fact that we compared synthetic SMCs in the control group to Jagged-induced synthetic SMCs. Thus, the variation in KI67 expression was a direct result of Notch activity. On the other hand, in the case of collagen, we compared synthetic and contractile cells with and without Jag1 coating. The observed changes in collagen expression might therefore also have been caused by differences in culture media used for synthetic and contractile cells. Nevertheless, the correlations obtained by comparing the data for each phenotype separately were very similar (Fig. 1B). This suggests that the changes in collagen expression can to a large extent be explained by changes in Notch activity. As Notch is not the only pathway regulating arterial G&R, the Notch stimulus functions were combined with phenomenological stimulus functions, in which all other mechanisms involved in arterial G&R were lumped together. This resulted in a combined stimulus function ϒ^*α*^ which can be substituted into Eq. (26) to regulate mass production:

(34) $${ϒ}^{\alpha }\left(\tau \right)={ϒ}_{N}^{\alpha }\left(\tau \right)+{ϒ}_{\sigma }^{\alpha }\left(\tau \right)+{ϒ}_{\tau_{w}}^{\alpha }\left(\tau \right)-2.$$

The phenomenological stimulus functions were formulated based on previous CMMs:

(35) $${ϒ}_{\sigma }^{\alpha }\left(\tau \right)=1+K_{\sigma }^{\alpha }\text{Δ}\sigma \left(\tau \right)$$

(36) $${ϒ}_{\tau_{w}}^{\alpha }\left(\tau \right)=1-K_{\tau_{w}}^{\alpha }\text{Δ}\tau_{w}\left(\tau \right)$$

where $K_{\sigma }^{\alpha }$ and $K_{\tau_{w}}^{\alpha }$ are constituent-specific gain parameters. Recall that Δ*σ* indicates the deviation in pressure- and axial force-induced intramural stress (Eq. (25)); Δ*τ* similarly gives the deviation in flow-induced wall shear stress *τ_w_*:

(37) $$\text{Δ}\tau_{w}\left(\tau \right)=\frac{\tau_{w}\left(\tau \right)-\tau_{w,o}}{\tau_{w,o}}=\frac{Q\left(\tau \right)r_{o}^{3}}{Q_{o}r^{3}}-1$$

with *Q* the cardiac output and *r* the luminal radius. The influence of Notch signaling on SMC contractility was not considered. When experimental data describing this influence become available, future studies might incorporate SMC activity in the CMM.

To explore the compatibility of the combined stimulus function, we investigated two different hypotheses for the definition of the scalar measure of the intramural stress, $\tilde{\sigma }$. For the first hypothesis, we assumed that the phenomenological stimuli ${ϒ}_{\sigma }^{\alpha }$ respond to changes in stress in both the circumferential and axial directions by taking the trace of the Cauchy stress, consistent with previous studies (Latorre and Humphrey 2018a, b)

(38) $$\tilde{\sigma }=\text{tr}\left(\sigma \right)=\sigma_{\theta \theta }+\sigma_{zz}$$

where a plane-stress state was inherently assumed, such that the radial component is very small compared to the other contributions: *σ_rr_* ≪ *σ_θθ_* and *σ_rr_* ≪ *σ_zz_*. For the second hypothesis, we assumed that only changes in the circumferential stress affected ${ϒ}_{\sigma }^{\alpha }$, consistent with the concept that SMCs mainly feel circumferential mechanical stimuli:

(39) $$\tilde{\sigma }=\sigma_{\theta \theta }$$

The vessel was modeled as a thin-walled cylinder, with the mean stresses in the circumferential (*σ_θθ_*) and axial (*σ_zz_*) directions given as a function of the blood pressure *P* and axial force on the vessel *f_z_*:

(40) $$\sigma_{\theta \theta }=\frac{Pr}{h}$$

(41) $$\sigma_{zz}=\frac{f_{z}}{\pi h\left(2r+h\right)}$$

with *h* the wall thickness. The initial value of the pressure, *P_o_*, was determined from the initial geometry, composition, and material properties, such that the vessel was in mechanical equilibrium in the reference configuration, and approximately equal to 14.4 kPa.

The deformation gradient tensor of the mixture was defined as:

(42) $$\text{F}\left(s\right)=\text{diag}\left(\lambda_{r}^{*},\lambda_{\theta }^{*},\lambda_{z}^{*}\right),$$

with $\lambda_{r}^{*},\lambda_{\theta }^{*}$, and $\lambda_{z}^{*}$ indicating the stretch ratios of the mixture from the reference configuration to the current configuration in radial, circumferential, and axial directions, respectively. The solution of the CMM was found by solving the Laplace Eqs. (40) and (41) for $\lambda_{\theta }^{*}$ and *f_z_*, at each time point with a step size of 0.1 days using a trust-region-dogleg algorithm. The hereditary integrals in Eqs. (4) and (5) were approximated using the Simpson’s rule for numerical integration, while the integral in Eq. (23) was estimated by multiplying the time-averaged degradation rate *k^a^* with the time step. The vessel was assumed not to deform in axial direction, resulting in the constant axial stretch $\lambda_{z}^{*}=1$. The radial stretch followed from the assumption of incompressibility: $\lambda_{r}^{*}=1/\left(\lambda_{\theta }^{*},\lambda_{z}^{*}\right)$. Subsequently, the wall thickness was calculated as

(43) $$h=\lambda_{r}^{*}h_{o},$$

and the luminal radius was calculated as

(44) $$r=\lambda_{\theta }^{*}\left(r_{o}+\frac{h_{o}}{2}\right)-\frac{h}{2}.$$

The system of ordinary differential equations ((27)−(30)) in the Notch model was solved for each SMC in the vessel wall individually, using an explicit time integration scheme with a time step of 0.05 h.

Parameter values for the CMM, the Notch model, and the coupling are given in Table 1. These values were in part derived from prior studies (Boareto et al. 2015; Bersi et al. 2017; Loerakker et al. 2018; Latorre et al. 2019; Irons et al. 2021) and in part determined in the present study (Sect. 3.2).

## 3 Results

### 3.1 The Notch model is compatible with the CMM when collagen production does not depend on axial stress

**Fig. 2:**
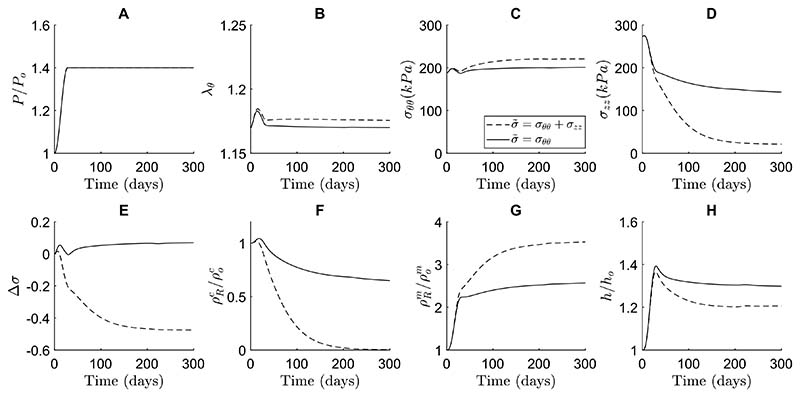
Predicted time course of blood vessel properties in response to hypertension for two definitions of the intramural stress $\left(\tilde{\sigma }\right)$, regulating the phenomenological stimulus functions ${ϒ}_{\sigma }^{\alpha }$, depending on either the circumferential stress $\left(\tilde{\sigma }=\sigma_{\theta \theta }\right)$, or both the axial and circumferential stress $\left(\tilde{\sigma }=\sigma_{\theta \theta }+\sigma_{zz}\right)$. The figure shows the results in terms of (**A**) blood pressure, (**B**) circumferential SMC stretch, (**C**) circumferential stress, (**D**) axial stress, (**E**) deviation in intramural stress, (**F**) referential mass density of collagen, (**G**) referential mass density of SMCs, and (**H**) wall thickness. The quantities in (**A, F, G** and **H**) were normalized to their original values at time *s* = 0

In previous CMMs, G&R was regulated by changes in the mechanical target variables of intramural stress and wall shear stress (Eqs. (25) and (37)). The addition of the Notch contribution in the current model introduced an additional target variable, namely circumferential strain of the SMCs. To understand the consequences of this addition, we first assessed the compatibility of the Notch contribution with the target variables in the CMM by comparing the two definitions of the intramural stress in Eqs. (38) and (39). As previous phenomenological CMMs have been tested in diverse contexts of vascular G&R, including hypertension (Humphrey 2021), we similarly simulated hypertensive aortic G&R (Fig. 2). The parameters for the Notch stimulus functions were based on the in vitro experiments (Fig. 1B), while the gain parameters of the phenomenological stimulus functions were derived from Latorre et al. (2019) (Table 1).

Surprisingly, adopting the first hypothesis $\left(\tilde{\sigma }=\text{tr}\left(\sigma \right)\right)$ resulted in a predicted total degradation of collagen (Fig. 2F, dashed line). This result can be explained by the following cascade of events. The increase in pressure (Fig. 2A) caused an initial increase in both *λ_θ_* (Fig. 2B) and Δσ (Fig. 2E), resulting in an increase in collagen (Fig. 2F) and smooth muscle content (Fig. 2G). In particular, the decrease in Notch activity in response to the increased *λ_θ_* (Eq. (32)) induced a large increase in SMC content (Fig. 2G). This caused a relatively steep increase in thickness (Fig. 2H), resulting in a decrease in the axial stress (Fig. 2D), following Eq. (41). This decrease in axial stress subsequently rendered Δ*σ* < 0 (Fig. 2E) which, in addition to the Notch response (Eq. 33), contributed to a reduction in collagen content via Eq. (35). As the collagen fibers were oriented diagonally, they contributed to the stress in axial direction. A decrease in the collagen content therefore reduced the axial stress further. As such, a positive feedback loop emerged in which the collagen content kept decreasing until it was completely removed from the tissue (Fig. 2F). This effect could not be compensated for by the increase in SMC content (Fig. 2G) as the SMCs were oriented only circumferentially. The removal of collagen from the tissue prevented the vessel from recovering its homeostatic mechanical state (Fig. 2B, E).

By contrast, when stress homeostasis for the phenomenological contribution was assumed to depend only on the circumferential stress $\left(\tilde{\sigma }=\sigma_{\theta \theta }\right)$, the coupled simulations predicted more realistic results (Fig. 2). With this definition, we saw a similar decrease in axial stress after an initial small increase (Fig. 2D). However, this decrease in axial stress did not reduce the collagen content because the axial stress no longer contributed to Δ*σ*. Therefore, a positive feedback loop between the decrease in axial stress and collagen content failed to emerge. Collagen content still decreased slightly because of the Notch stimulus function (Eq. (33)), which lowered collagen production upon a reduction in Notch activity in response to the increase in *λ_θ_* (Fig. 2B). Nevertheless, the large increase in SMC content, partly caused by Notch (Eq. (32)), allowed the vessel to reach a state much closer to the original mechanical homeostasis (Fig. 2B, E). Overall, these simulations suggest that, with the current assumptions for the Notch model as informed by the in vitro data, the two models are compatible only when collagen production does not depend on axial stress. This assumption was thus adopted for all subsequent simulations.

### 3.2 A computational model combining a Notch stimulus with a phenomenological stimulus can capture in vivo arterial G&R in hypertension

**Fig. 3:**
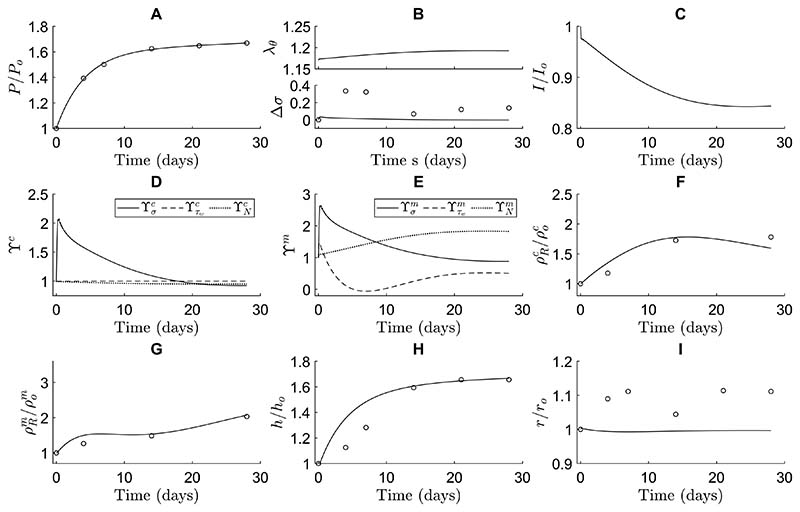
The computational framework can mimic arterial G&R in response to hypertension. The predicted time course of blood vessel properties is shown in terms of (**A**) blood pressure, (**B**) circumferential SMC stretch (top) and deviation in intramural stress (bottom), (**C**) NICD content, averaged over the SMCs, indicating Notch activity, (**D**) stimulus functions for collagen, (**E**) stimulus functions for SMCs, (**F**) referential mass density of collagen, (**G**) referential mass density of SMCs, (**H**) wall thickness, and (*I*) luminal radius. All quantities were normalized to their original values at time *s* = 0, except in (**B, D** and **E**). The open circles represent the in vivo data by Bersi et al. (2017). The individual contributions of intramural stress (solid lines), wall shear stress (dashed lines), and Notch signaling (dotted lines) to the stimulus functions for collagen and SMCs are shown in (**D, E**)

Next, we analyzed whether the present framework combining Notch-driven and phenomenological stimulus functions can capture in vivo arterial responses to hypertension. As in the previous simulations (Fig. 2), the parameters of the Notch stimulus functions were derived from the in vitro experiments (Fig. 1B). Parameters for the phenomenological stimulus functions were determined via regression of published data on hypertensive remodeling of the murine infrarenal abdominal aorta (Bersi et al. 2017) and subsequent analysis (Latorre et al. 2019). First, a pressure profile was imposed similar to the one observed experimentally (Fig. 3A). The gain parameters $K_{\sigma }^{\alpha }$ and $K_{\tau_{w}}^{\alpha }$, with *α* = *m, c*, from Eqs. (35) and (36) were then determined from experimental data on evolving collagen mass density, SMC mass density, wall thickness, and luminal radius (Fig. 3F-I). The normalized SMC and collagen mass densities were calculated from the reported area fractions of SMCs and collagen (Bersi et al. 2017) and the cross-sectional areas of the media in the loaded state (Latorre et al. 2019).

The combined model was able to capture the experimentally observed changes in thickness and constituent mass densities in response to hypertension (Fig. 3). The elevated blood pressure (Fig. 3A) increased circumferential SMC stretch and intramural stress (Fig. 3B). These increases in stretch led to a decreased expression of Notch receptors and Jagged ligands (Eqs. (27) and (28)). Consequently, Notch activity was reduced, represented by a lower NICD content (Fig. 3C), which gave rise to an increase in SMC proliferation and a decrease in collagen synthesis, dictated by the Notch stimulus functions (Fig. 3D and E) that were informed by the in vitro experiments (Eqs. (32) and (33)). Regarding the phenomenological component of the model, the increase in intramural stress caused an increase in both SMC proliferation and collagen synthesis (Fig. 3D and E), in accordance with Eq. (35). The combination of all stimuli (Eq. (34)) resulted in an increase in collagen and SMC mass densities (Fig. 3F and G), thereby causing an increase in wall thickness (Fig. 3H). The thickening of the wall lowered the stresses (Eqs. (40), (41)) and reduced the pressure-induced increase in strain (Fig. 3B), ultimately stabilizing wall thickness at a new equilibrium value (Fig. 3H). Despite this equilibrium in thickness, the individual stimulus functions did not reach a value of one (Fig. 3D, E), due to the different target variables driving these stimulus functions (i.e., SMC strain, intramural stress, and wall shear stress). As the composition of the tissue, and thereby its mechanical properties, can change, these target variables may not all be fully restored to their original values, illustrated by *λ_θ_* in Fig. 3B. Recall, therefore, that the term homeostasis implies “similar to” rather than “the same as,” hence homeostatic quantities tend to be restored toward, but not precisely to, original values. This indicates that arterial adaptation may seek to find a compromise in maintaining these target variables. In summary, these simulations demonstrate the ability of the combined model, including a mechanistic description of mechano-sensitive Notch signaling based on independent in vitro experiments, to predict key features of in vivo arterial adaptation to hypertension.

The model was nevertheless not able to predict the increase in internal radius observed in the experiments (Fig. 3I). This may have been due, in part, to the lack of inflammation in the model, which was present at low levels in the experiments (Bersi et al. 2017). Given that previous phenomenological CMMs have been able to predict small increases in radius even without considering inflammation (Valentin et al. 2009, Irons et al. 2021), these findings together suggest that adding explicit Notch signaling to the model reduced the sensitivity of adaptations of radius. A possible explanation for this is that the prescribed Notch signaling attempted to restore SMC stretch (not previously considered) to its original value while radius is typically thought to be regulated primarily by changes in wall shear stress (Humphrey 2008). Although wall shear stress is known to influence Jagged and Notch in endothelial cells (Mack et al. 2017; Driessen et al. 2018), this phenomenon was not included in the current Notch model. In addition, active SMC stress, not modeled here, affects the radius and is partly controlled by Notch signaling. Future efforts should therefore investigate whether adding the effects of shear stress on Notch in endothelial cells and active SMC stress regulated by Notch could improve the predicted evolution of the radius.

The combined model further predicted changes in intramural circumferential stress that were lower than those in the experimental data set (Fig. 3B). This was likely a consequence of the relatively high gain parameters of the phenomenological stimulus functions (Table 1), rendering the model very sensitive to changes in pressure. As a result, even small increases in intramural stress due to hypertension caused large increases in collagen production (Fig. 3D) and SMC proliferation (Fig. 3E). Related to this, the high gain parameters may also explain the slight overestimation of the predicted thickness in the early stages of adaptation (Fig. 3H) as they were responsible for the rapid increase in SMC proliferation and collagen production (Fig. 3D-G) in response to the relatively steep increase in pressure during the first few days.

Taken together, our results demonstrate that the combination of specific mechanistic (Notch) and phenomenological (remaining) stimuli can capture the increase in thickness and constituent mass densities observed in arterial hypertension. Nevertheless, the model could be expanded in future to refine the Notch model with the aim of improving predictions of evolving radius and intramural stress.

### 3.3 Notch mechano-sensitivity mainly contributes to smooth muscle cell proliferation during adaptation to hypertension

To gain more insight into the roles of Notch signaling in arterial adaptation to hypertension, we compared predictions of three related models. The first was the combined model, including both the phenomenological and Notch stimulus functions; the second was phenomenological only, with the Notch contribution deactivated $\left({ϒ}_{N}^{\alpha }=1\right)$; the third included only the Notch stimulus functions, without the phenomenological contribution $\left({ϒ}_{\sigma }^{\alpha }={ϒ}_{\tau_{w}}^{\alpha }=1\right)$. The phenomenological functions ${ϒ}_{\sigma }^{\alpha }$ depended on the circumferential stress only (Eq. 39), even when the Notch contribution was deactivated, to allow for consistent comparisons among the different contributions to ϒ^α^ within this study (recall Sect. 3.1). This, together with the newly fitted gain parameters (Sect. 3.2), explains the different predictions given by the phenomenological models described here and in Latorre et al. (2019), in particular for intramural stresses and luminal radius.

**Fig. 4:**
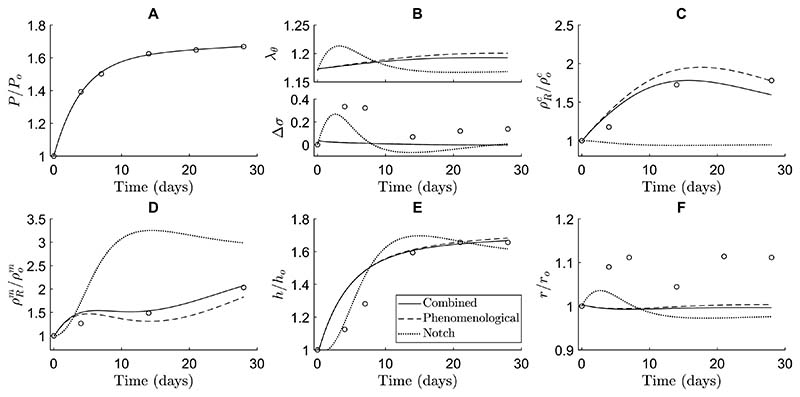
The contribution of Notch signaling to arterial adaptation blood pressure, (**B**) circumferential SMC stretch (top) and deviation to hypertension is revealed by comparing three different models: a in intramural stress (bottom), (**C**) referential mass density of collagen, phenomenological-only model (dashed lines), in which G&R is regu-(**D**) referential mass density of SMCs, (**E**) wall thickness, and (**F**)lated only by the phenomenological stimulus functions; a Notch-only luminal radius. All quantities were normalized to their original values model (dotted lines), in which G&R is regulated only by the Notch at time *s* = 0 except in (**B**). The open circles represent in vivo data by stimulus functions; and the combined model (dashed lines), in which Bersi et al. (2017)G&R is regulated by both. The results are shown in terms of (**A**) blood pressure, (**B**) circumferential SMC stretch (top) and deviation in intramural stress (bottom), (**C**) referential mass density of collagen, (**D**) referential mass density of SMCs, (**E**) wall thickness, and (**F**) luminal radius. All quantities were normalized to their original values at time *s* = 0 except in (**B**). The open circles represent in vivo data by Bersi et al. (2017)

These simulations showed that Notch signaling contributed primarily to the proliferation of SMCs. The Notchonly model predicted a substantial increase in SMC mass density (Fig. 4D) and a small decrease in collagen mass density (Fig. 4C) due to an initial increase in SMC stretch (Fig. 4B). The increase in SMC proliferation (Fig. 4D) was in line with the in vivo data (Bersi et al. 2017) and contributed to the thickening of the arterial wall (Fig. 4E). This thickening lowered SMC stretch (Fig. 4B), revealing a negative feedback mechanism driving Notch-regulated G&R, aimed at restoring the original SMC stretch levels. This role of Notch in SMC proliferation was also demonstrated in the combined model by the value of the Notch stimulus function for SMC: ${ϒ}_{N}^{m}>1$ (Fig. 3E). The phenomenological stimulus function driven by intramural stress, ${ϒ}_{\sigma }^{m}$, was synergistic with Notch and also contributed to SMC proliferation, especially at early time points (Fig. 3E).

The simulations further revealed that mechano-sensitive Notch signaling could explain arterial growth, but not remodeling, in response to hypertension. Remarkably, the model with only Notch stimulus functions, informed by independent in vitro data, captured both the new equilibrium thickness and its temporal profile reasonably well (Fig. 4E). This suggests that the negative feedback mechanism between SMC stretch and Notch-regulated G&R may be an important factor in arterial thickening in response to hypertension. Yet, the Notch-only model did not capture the experimentally observed changes in constituent mass densities (Fig. 4C and D). In fact, as the hypertension-induced increase in SMC stretch reduced Notch activity and consequently downregulated collagen production (Eq. (33)), collagen mass density was underestimated (Fig. 4C) and SMC mass density was overestimated (Fig. 4D) in the attempt to reach the wall thickness (Fig. 4E) needed for restoring the original SMC stretch (Fig. 4B). Simulations with the phenomenological-only model showed that further contributions were needed to capture more accurately the experimentally observed changes in constituent mass densities. In particular, the lack of Notch-driven collagen production was compensated for by an increase in collagen production, driven by the phenomenological stimulus functions (Figs. 4C and 3D).

Taken together, these simulations suggest that Notch mainly contributes to SMC proliferation in hypertensive vessels. Thus, Notch mechano-sensitivity might be key for arterial growth in response to hypertension, but other mechanisms are needed to fully capture remodeling.

### 3.4 Notch interventions can alter both arterial geometry and composition in hypertension

The simulations in Fig. 3 and 4 demonstrated that our combined model captured in vivo arterial adaptations to hypertension well, similar to existing phenomenological CMMs (Valentin et al. 2009, Latorre et al. 2019). The main benefit of the combined model compared to phenomenological-only models is that it enabled us to examine potential effects of interventions to the Notch pathway on arterial G&R. To further this understanding, here we investigated potential effects resulting from the addition of soluble and immobilized Jagged ligands to SMCs in a hypertensive arterial wall. Such interventions have previously been suggested as treatment for vascular diseases (Xiao et al. 2013; Caolo et al. 2011) or in the context of vascular regeneration (Carlson et al. 2007; Zohorsky and Mequanint 2021; Karakaya and Van Asten et al. 2022a, Tiemeijer and Sanlidag 2022b).

**Fig. 5:**
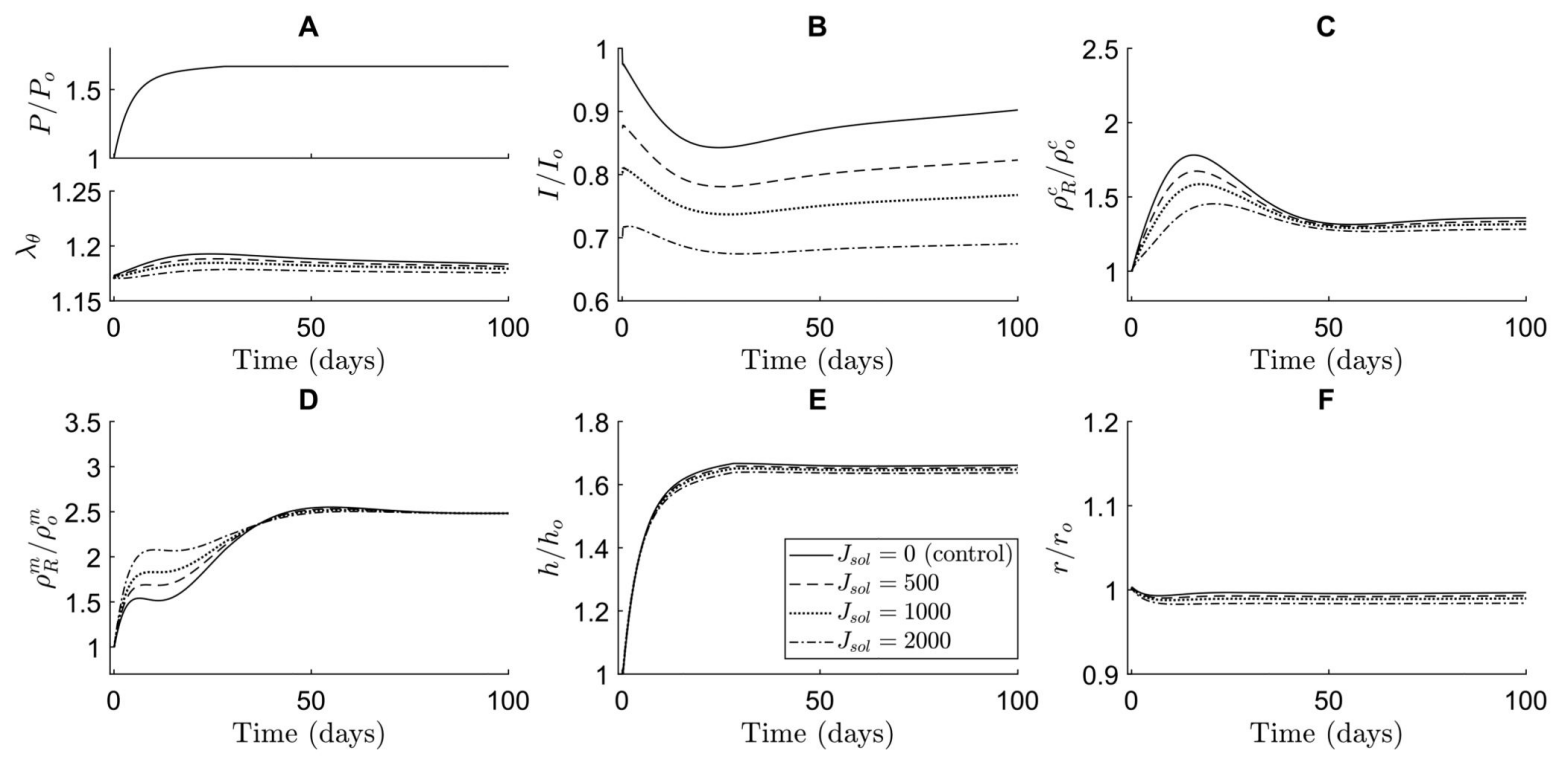
The simulations indicate that soluble Jagged ligands cause an increase in SMC proliferation and decrease in collagen density. These effects are dose-dependent and especially evident in the short-term. The effects of different concentrations of immobilized Jagged are compared in terms of (**A**) blood pressure (top) and circumferential SMC stretch (bottom), (**B**) NICD content, averaged over the SMCs, indicating Notch activity, (**C**) referential mass density of collagen, (**D**) referential mass density of SMCs, (**E**) wall thickness, and (**F**) luminal radius. All quantities were normalized to their original values at time *s* = 0 except the circumferential SMC stretch ((**A**) bottom). The values of *J_sol_* represent the constant number of soluble Jagged ligands available to each SMC, where *J_sol_* = 0 is the control simulation without soluble Jagged ligands

In our simulations, soluble Jagged ligands primarily affected the transient G&R during hypertensive stimulation reducing the long-term equilibrium thickness and collagen mass density (Fig. 5). These soluble Jagged ligands were assumed to be able to bind to Notch receptors without activating them. Hence, they prevented cell-bound ligands from binding to and activating the Notch receptors, thus lowering the NICD content compared to the control simulation without soluble Jagged ligands (Fig. 5B). Interestingly, this decrease in Notch activity caused a temporary increase in SMC proliferation without affecting the final equilibrium SMC mass density (Fig. 5D). It also resulted in a general decrease in collagen mass density, especially in the short term (Fig. 5C). Due to the temporal increase in SMC density and decrease in collagen density, the thickness over time was only marginally smaller compared to the control simulation (Fig. 5E). These simulations suggested further that this process is dose-dependent, with a higher concentration of soluble Jagged ligands resulting in more pronounced effects.

**Fig. 6:**
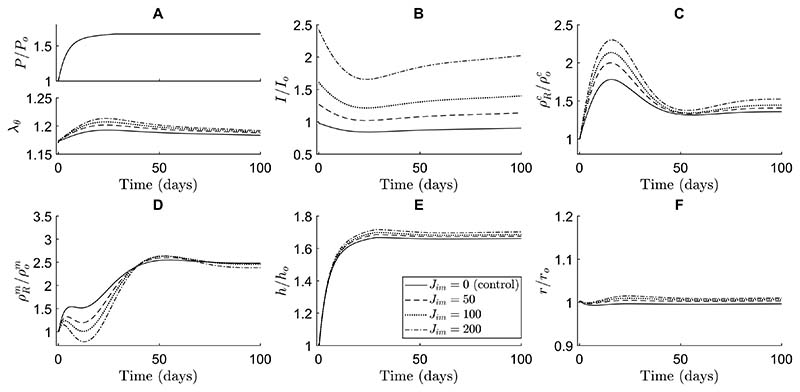
The simulations indicate that immobilized Jagged ligands cause a decrease in SMC proliferation and increase in collagen density. These effects are dose-dependent and more pronounced in the short-term, although also still present in the long-term. The effects of different concentrations of immobilized Jagged are compared in terms of (**A**) blood pressure (top) and circumferential SMC stretch (bottom), (**B**) NICD content, averaged over the SMCs, indicating Notch activity, (**C**) referential mass density of collagen, (**D**) referential mass density of SMCs, (**E**) wall thickness, and (**F**) luminal radius. All quantities were normalized to their original values at time *s* = 0 except the circumferential SMC stretch ((**A**) bottom). The values of *J_im_* represent the constant number of immobilized Jagged ligands available to each SMC, where *J_im_* = 0 is the control simulation without immobilized Jagged ligands

Immobilized Jagged ligands had the opposite effect compared to their soluble counterparts in the simulations. These ligands were assumed to bind and activate Notch receptors, thereby increasing the NICD levels (Fig. 6B). This caused a higher collagen production (Fig. 6C) and lower SMC proliferation (Fig. 6D), especially in the early stages of adaptation. The effects of immobilized Jagged ligands on both collagen and SMC mass density remained in the long term, albeit less pronounced as time progressed (Fig. 6C and D). The arterial wall was predicted to become slightly thicker as a result of the changes in constituent mass densities (Fig. 6E). Again, the extent of these effects was shown to depend on the concentration of Jagged ligands (Fig. 6). These results also suggest that changes in Notch signaling do not necessarily translate directly into intuitive changes in geometry. A decrease in Notch activity, for example, may be expected to cause thickening due to SMC proliferation, but in our simulations resulted in a small decrease in thickness due to a substantial decrease in collagen content.

Interestingly, our simulations predicted that Notch-mediated G&R was much more sensitive to immobilized than to soluble Jagged ligands. A concentration of soluble Jagged ligands of about an order of magnitude higher than immobilized Jagged ligands was needed to achieve similar changes in constituent mass densities (Figs. 5 and 6). This finding may indicate that there was a large number of Notch receptors compared to ligands on the SMCs. In that case, the soluble Jagged ligands occupying Notch receptors would not have a large effect because there would be plentiful free Notch receptors left for cell-bound ligands to activate. On the other hand, the immobilized Jagged ligands would find a lot of potential Notch receptors to which to bind, maximizing their effect. Furthermore, the changes caused by external Jagged primarily occurred in the early phases of adaptation. This was caused by other mechanisms, here captured in the phenomenological stimulus functions, compensating for the effects of external Jagged in the long term.

In summary, and in contrast to a purely phenomenological approach, the combined model also enabled us to investigate potential effects of interventions to the Notch pathway on arterial G&R. Our model predicted that Notch-mediated G&R is more sensitive to immobilized than to soluble Jagged ligands, and that introducing these external Jagged ligands primarily affected the arterial wall composition in the short-term.

## 4 Discussion

To advance our understanding of the role of Notch signaling in hypertensive remodeling, we developed a multiscale computational framework by coupling a model for mechanosensitive Notch signaling to a CMM of arterial mechanics and G&R. The deposition and degradation of collagen and the proliferation of SMCs were thereby described by a combination of phenomenological and mechanistic contributions. The latter were based on mechano-sensitive Notch signaling and informed by independent in vitro data. The phenomenological contributions were mediated by stress homeostasis and accounted for mechanisms and pathways other than Notch. The combined model was able to capture evolving changes in abdominal aortic thickness and wall composition in response to induced hypertension in a common mouse model. Importantly, our simulations suggested that Notch mechano-sensitivity contributes primarily to such thickening by increasing SMC proliferation. The model also enabled the investigation of interventions in the Notch pathway: simulations showed that external Jagged ligands may alter arterial wall composition and thickness in hypertensive arteries.

The combined model presented here provides a step forward in integrating mechano-sensitive Notch signaling and arterial G&R in a computational framework. While many existing G&R frameworks have adopted a fully phenomenological description of tissue production and removal (e.g., Baek et al. 2006, 2007, Valentin et al. 2009, 2011, Latorre et al. 2019; Latorre and Humphrey 2020), our combined model includes the contribution of one key cellular mechanism. An important advantage of this model is that it can address the role of Notch in arterial G&R and simulate both pathological mutations and pharmacological interventions. Similarly, other models have included cellular mechanisms underlying mechano-regulated G&R, such as TGF-β, interleukins, PDGF, and MMPs (Aparicio et al. 2016; Marino et al. 2017; Keshavarzian et al. 2018; Khosravi et al. 2020; Irons and Humphrey 2020). Some of them have considered one-way feedback between tissue mechanics and cellular mechanisms (Khosravi et al. 2020; Irons and Humphrey 2020), without input from the cell behavior back to the tissue’s mechanical state, thereby limiting the application of these models to the analysis of short-term behavior. Here, by capturing the full bi-directional feedback, we can predict long-term G&R and the response to sustained perturbations. A few recent models have also considered multiscale feedback, as, for example, in the context of aneurysm development (Aparicio et al. 2016), tissue engineering (Keshavarzian et al. 2019), and arterial adaptation (Irons et al. 2021, 2022). None of these models so far focused on Notch signaling despite the well-recognized role of Notch in vascular morphogenesis, homeostasis, and pathogenesis (Iso et al. 2003; Gridley 2007; Del Monte et al. 2011; Baeten and Lilly 2017; Mašek and Andersson 2017; Li and Kong 2020; Ristori et al. 2021; Karakaya et al. 2022a). Thus, our model adds to the development of more mechanistic descriptions of G&R by considering Notch signaling, one of the key mediators of vascular homeostasis.

The combination of a mechanistic and a phenomenological approach in our model enabled us to isolate the Notch pathway and analyze its contribution to changes in geometry and composition compared to other mechanisms. Despite its important role, Notch signaling contributes only partially to the general G&R process, acting in concert with many other pathways such as angiotensin, TGF-β, integrins, WNT, and VEGF, to name a few (reviewed in LaFoya et al. 2016). Here, we therefore used a parallel phenomenological contribution to account for any mechanisms other than Notch. Notwithstanding the advantage of focusing on a single pathway, an important limitation of this approach is that the phenomenological and Notch contributions were treated separately as independent factors. Crosstalk between Notch and other pathways that were represented phenomenologically was not considered. This crosstalk can result in complex responses that may be synergistic with or antagonistic to the individual behavior of the isolated Notch pathway. Whereas this approach can be contrasted with other models wherein multiple key pathways were considered together (Khosravi et al. 2020; Irons and Humphrey 2020), the present results suggest that it would be of interest to consider many different pathways individually, as done herein for Notch, to begin to understand better the complex interactions across pathways, noting that sub-pathway analyses have similarly proven insightful (Estrada, et al. 2021).

Previous computational studies have also investigated the role of mechano-sensitive Notch signaling in the context of arterial G&R. For example, simulations suggested that Notch mechano-sensitivity may be key in establishing a homeostatic thickness in healthy arteries (Loerakker et al. 2018). Subsequently, Notch mechano-sensitivity was also implicated in arterial thickening in response to hypertension (Van Asten and Ristori et al. 2022). In the present study, this finding was confirmed (Fig. 3H), and we were able to predict the role of Notch mechano-sensitivity in more detail, by capturing changes in arterial wall composition in addition to geometry. This more detailed analysis highlighted that Notch mainly contributes to hypertensive G&R by promoting SMC proliferation. While the model suggested that Notch can explain hypertensive thickening reasonably well (Fig. 4E), it did not capture the reported changes in composition (Fig. 4C, D). Particularly, while the decrease in collagen production in response to a reduction in Notch activity (Fig. 1B, 3D, 4C) is consistent with previous in vitro results (Lilly and Kennard 2009; Lin and Lilly 2014), an increase in collagen content is expected in hypertensive arteries (Bersi et al. 2017). This suggests that other mechanisms are also involved in hypertensive aortic remodeling, demonstrated in the model by the fact that the phenomenological contribution was necessary to capture the experimentally observed changes in constituent mass densities (Figs. 3D, E, 4C, D). Caution should therefore be used when modeling just one pathway, as other mechanisms need to be accounted for as well. Overall, the simulations revealed that Notch may influence vessel wall thickness in response to hypertension mainly by regulating SMC proliferation, while collagen production might depend on other mechanisms.

The choice of target variables for mechanical homeosta sis is still debated (Cyron and Humphrey 2017; Eichinger et al. 2021), and may be context dependent. Our simulations revealed that the choice of the target variables was crucial to achieve realistic adaptive responses to hypertension when the phenomenological contribution was combined with our Notch signaling model (Fig. 2). In particular, there appears to be a need for consistency across scales—assuming that Notch is mechano-regulated by circumferential stretch alone required us to assume that the phenomenological response was similarly regulated by circumferential, not biaxial, stress (Fig. 2). Although SMCs, key regulators of vascular G&R (Owens et al. 2004), are oriented mainly in the circumferential direction, they are yet subjected to biaxial loading (Caulk et al. 2019) and vascular adaptations tend to depend strongly on changes in the axial direction (Humphrey et al. 2009). There is, therefore, a pressing need for more data on the biaxial responsiveness to guide further model development across tissue and cell scales. In addition, to facilitate the comparison between computational and in vivo data (Bersi et al. 2017), here the target variables were calculated at systolic pressure, while previous analysis suggested that sensitivity of Notch to mean pressure might result in more realistic predictions (Van Asten and Ristori et al. 2022). This choice, given the higher systolic versus mean pressure, may have exacerbated the response of Notch to hypertension, thereby enhancing the Notch-mediated wall thickening, resulting in the decrease in axial stress that led to the positive feedback loop causing the unrealistic complete removal of collagen (Fig. 2).

Combining different target variables also indicated that a wall thickness equilibrium can be achieved even when the individual target variables are not restored to their original values (Fig. 3). In particular, our model includes a stress-related target variable for the phenomenological contribution and a strain-related target variable for Notch signaling. The simulations revealed that the adaptation response to hypertension may not be able to restore both to their original values (Fig. 3B), as the composition, and thereby the mechanical properties, of the tissue can change during adaptation. This suggests that a compromise among the different individual targets may be found (e.g., via the combined stimulus function in Eq. (34)) or, possibly, that in vivo vessels aim to restore a different, yet unidentified, target variable. These conclusions were reached by assuming that Notch is sensitive to strain, based on previous experiments (Morrow et al. 2005; Loerakker et al. 2018). However, strain was chosen in these previous studies as it can directly be measured in vitro, which does not rule out that Notch may be sensitive to stress instead, similar to the phenomenological contribution. Nevertheless, given the similarity between computational and in vivo data, our results show that incorporating multiple biological mechanisms regulated by different target variables is a feasible approach for G&R frameworks.

Compared to previous phenomenological models (e.g., Latorre et al. 2019), our model was less able to predict the time course of changes in intramural stress (Fig. 3B) and luminal radius (Fig. 3I) in response to hypertension. This limitation may be a result of the approach chosen for the correlation between Notch activity and tissue formation. In particular, this correlation was determined by fitting the Notch stimulus functions (Eqs. (32) and (33)) to independent in vitro data (Fig. 1B). While these in vitro data may reveal general qualitative trends about up- or downregulation of certain genes in response to Notch, they may be considered as only an estimation of the quantitative behavior expected in vivo. A possible alternative approach would be fitting the parameters of the Notch stimulus functions to in vivo data. However, given that pure phenomenological models can already capture G&R in response to hypertension very well, fitting the parameters without additional data demonstrating the fundamental role of Notch in this context would incorrectly minimize the contribution of the Notch stimulus functions in hypertension. Detailed data on the effects of Notch manipulations (e.g., knock-outs) on changes in arterial geometry and composition would therefore be required. Unfortunately, existing data are currently insufficient, as they often report only changes in thickness and not wall composition or associated mechanical properties (Boulos et al. 2011; Ragot et al. 2016; Dave et al. 2022). This highlights a clear need for more quantitative data on the role of Notch in in vivo remodeling to obtain stronger correlations and further refine the model assumptions and parameters.

The in vitro experiments informing the present combined model showed that a reduction in Notch3 activity promoted SMC proliferation (Fig. 1B). This is not in line with previous findings showing an increase in SMC proliferation due to Notch1 activity in the rat pulmonary artery (Havrda et al. 2006), mouse aorta (Li et al. 2009), and human coronary artery (Morrow et al. 2010), and due to Notch3 activity in the rat aorta (Campos et al. 2002; Sweeney et al. 2004). Furthermore, while increased proliferation is a common feature of hypertension, it was found to be accompanied by higher, rather than lower, levels of Notch activity in pulmonary artery hypertension (Li et al. 2009; Qiao et al. 2012; Xiao et al. 2013; Morris et al. 2019). Notch is well-known to show differential outcomes depending on context, location, cell type, and receptor-ligand pair (Boucher et al. 2012; Stassen et al. 2020; Karakaya et al. 2022b). Our in vitro data correlated Notch3 expression in human coronary artery SMCs to their proliferation. Thus, the difference in Notch receptor and cell type compared to prior studies may explain some of the described inconsistencies. The predictions from the current model may therefore be less accurate for other cell types or locations in the vasculature. This re-emphasizes the need for more context-specific data relating Notch to tissue formation. Such data could easily be incorporated in the current framework by adapting only the Notch stimulus functions (Eqs. (32) and (33)).

To investigate the potential effects of Notch interventions on hypertensive aortic G&R, we simulated the addition of soluble and immobilized Jagged ligands (Fig. 5, 6). In case of immobilized Jagged, our model predicted a decrease in SMC proliferation and an increase in collagen production due to higher Notch activity, which is consistent with human in vitro and in vivo experimental findings in keratinocytes, dental pulp cells, and periodontal ligament cells (Beckstead et al. 2009; Manokawinchoke et al. 2017; Suwanwela et al. 2019). On the other hand, soluble Jagged ligands in the model caused an increase in SMC proliferation due to a reduction in Notch activity, which disagrees with previous in vitro results in human coronary artery SMCs and in vivo results in the murine pulmonary artery and in a murine vein graft (Caolo et al. 2011; Xiao et al. 2013; Zhou et al. 2015). The agreement between simulations and experiments in case of immobilized Jagged and disagreement in case of soluble Jagged may suggest that other mechanisms of the Notch pathway are involved. For example, interactions between soluble Jagged and other receptors not currently present in the model, such as Notch2, might compensate for the decrease in Notch3 activation due to soluble Jagged, given the known compensatory roles of Notch2 and Notch3 in SMCs (Wang et al. 2012; Baeten and Lilly 2017).

The ability of the model to predict the consequences of Notch interventions may have applications in vascular pharmacology and regenerative medicine. For example, inhibiting Notch via soluble Jagged ligands or γ-secretase inhibitors has been suggested as a treatment for both pulmonary arterial hypertension (Li et al. 2009; Xiao et al. 2013) and extracranial vascular malformations (Davis et al. 2018). Notch inhibition has also been shown to inhibit neointima formation (Caolo et al. 2011) and regress abdominal aortic aneurysms (Sharma et al. 2019). By predicting such interventions (Figs. 5 and 6), the model may serve as a tool for exploring the therapeutic potential of Notch and guiding future experiments. In the context of vascular regeneration, Notch signaling has also been identified as a possible target (Carlson et al. 2007; Zohorsky and Mequanint 2021; Karakaya and Van Asten et al. 2022a, Tiemeijer and Sanlidag 2022b). For example, Jagged ligands can be immobilized to biomaterials (Putti et al. 2019a, b), a method that has been used to control the differentiation of keratinocytes (Beckstead et al. 2009), dental pulp cells (Manokawinchoke et al. 2017) and coronary artery SMCs (Zohorsky et al. 2021). Immobilizing ligands to scaffolds may similarly aid in the improvement of tissue-engineered vascular grafts. Our simulations (Figs. 5, 6) imply that this would primarily affect the early composition of these grafts and thereby their mechanical properties, which might help to prevent cases of graft dilatation (Tara et al. 2015; Yang et al. 2016) or stenosis (Koobatian et al. 2016; Khosravi et al. 2020). Combined models may aid in systematic and efficient optimization of scaffold design to minimize the need for expensive and time-consuming trial-and-error experiments.

In conclusion, this study represents another step forward in vascular simulation by including effects of mechano-sensitive Notch signaling in a computational G&R framework. The mechanistic description led to the suggestion that Notch contributes primarily to SMC proliferation in aortic adaptation to hypertension and that other mechanisms are necessary to fully capture remodeling. In addition, external Jagged ligands can affect the short-term composition of the arterial wall. Future studies should seek to improve the model further by implementing context-specific in vivo data on Notch signaling and by considering effects of other pathways and their crosstalk with Notch. The model can serve as a time- and cost-efficient tool to inspire new treatment strategies for vascular diseases or to optimize current methods in vascular regenerative medicine.

## Figures and Tables

**Table 1:** Parameter values for the combined model. The parameter values for the CMM were based on measurements in Bersi et al. (2017) and subsequent regression performed in Latorre et al. (2019) for the murine infrarenal abdominal aorta. Mass fractions $\Phi_{\text{o}}^{\alpha }$ were derived from Irons et al. (2021) and the gain parameters $K_{\sigma }^{\alpha }$ and $K_{\tau_{\text{w}}}^{\alpha }$ used in Figs. 3, 4, 5, 6 were determined via regression in Sect. 3.2. The parameter values for the Notch model were based on previous models in Boareto et al. (2015) and Loerakker et al. (2018)

Constrained mixture model	
*c^e^*	114 kPa
$\left[c_{1}^{c},c_{2}^{c}\right]$	[450 kPa, 3.51]
$\left[c_{1}^{m},c_{2}^{m}\right]$	[343 kPa, 1.23]
$\left[G_{r}^{e},G_{\theta }^{e},G_{z}^{e}\right]$	[$1/G_{\theta }^{e}G_{z}^{e}$, 1.96, 1.73]
$\left[G_{\theta }^{c},G_{z}^{c}\right]$	[1.17, 1.2]
$G_{\theta }^{m}$	1.17
*h_o_*	0.032 mm
$\left[k_{o}^{c},k_{o}^{m}\right]$	[1/10, 1/10] day^−1^
$\left[K_{\sigma }^{c},K_{\tau_{w}}^{c}\right]$	[0.55, 1.65] (Fig. 2) [32.6, 0] (Figs. 3, 4, 5, 6)
$\left[K_{\sigma }^{m},K_{\tau_{w}}^{m}\right]$	[0.473, 1.41] (Fig. 2) [50, 50] (Figs. 3, 4, 5, 6)
*M_o_*	16
*P_o_*	14.4 kPa
*r_o_*	0.417 mm
*ρ*	1050 kg/m^3^
*ϕ*	30.7°
$\left[\Phi_{o}^{e},\Phi_{o}^{c},\Phi_{o}^{m}\right]$	[0.079, 0.595, 0.326]
Notch model	
[*A_N_, A_J_*]	[−5.79, −4.17]
*I*_0_	200
[*k_t_, k_c_*]	[2.5, 5.0] 10^−5^ h^−1^
[*N_pr_, J_pr_, D_pr_*]	[1400, 1600, 100]
[*p_N_, p_J_, p_D_*]	[2.0, 5.0, 2.0]
[*γ, γ_I_*]	[0.1, 0.5]
[Λ_*N*_, Λ_*J*_, Λ_*D*_]	[2.0, 2.0, 0.0]

## Data Availability

All data and computational codes are available at https://doi.org/10.4121/22040729.
